# Excessive preoccupation of having a serious illness in medicine residents

**DOI:** 10.1192/j.eurpsy.2025.1011

**Published:** 2025-08-26

**Authors:** A. Mhalla, A. Ben Haouala, I. Betbout, A. Haj Salah, B. Amamou, F. Zaafrane

**Affiliations:** 1 Laboratory research LR05ES10, University of Monastir, Monastir, Tunisia

## Abstract

**Introduction:**

Excessive preoccupation of having a serious illness (EPHSI) or illness anxiety disorder is frequently reported by doctors and medical students

**Objectives:**

Our objectives were to estimate the frequency of EPHSI in a population of medicine residents in Tunisia and to determine the factors associated with this pathology.

**Methods:**

This was a cross-sectional study via internet, carried out among 404 medicine residents practicing in Tunisia during the period of November and December 2020 and January 2021.

The information was collected using a pre-designed questionnaire, edited using the Google site and disseminated via social networks. This questionnaire was inspired by the Health Anxiety Questionnaire, the MSD score, and the DSM-5 criteria for illness anxiety disorder

**Results:**

In the end of the study period, 404 medicine residents responded to the questionnaire. The mean age was 27.4 years, the sex ratio was 0.47. Residents who fulfilled the DSM5 criteria of anxiety illness disorder represented 33.7% of the total group.

The most preoccupying diseases were neoplastic diseases (40.8%), neurodegenerative disorders (14.3%), and psychiatric disorders (10%).

EPHSI was associated to several sociodemographic, anamnestic and psychological factors

**Conclusions:**

The establishment of psychological support systems for students, emphasizing coping and stress management techniques, should be implemented to prevent the EPHSI.

**Disclosure of Interest:**

None Declared

